# Integrated Virtual Screening for Anti-Caries Compounds from Neem: Dual-Target Inhibition of Biofilm Formation and Bacterial DNA Replication

**DOI:** 10.3390/biomedicines13092202

**Published:** 2025-09-08

**Authors:** Oluwaseun E. Agboola, Oluwatimileyin Agboola, Zainab A. Ayinla, Samuel S. Agboola, Oluranti E. Olaiya, Oluwatoyin M. Oyinloye, Omotola M. Fajana, Olajumoke Tolulope Idowu, Olaposi I. Omotuyi, Olutosin S. Ilesanmi, Babatunji E. Oyinloye

**Affiliations:** 1 Institute for Drug Research and Development, Bogoro Research Centre, Afe Babalola University, P.M.B 5454, Ado-Ekiti 360001, Nigeriaolaposi.omotuyi@abuad.edu.ng (O.I.O.); babatunjioe@abuad.edu.ng (B.E.O.); 2 Damsem Scientific Laboratory and Research, Ado-Ekiti 360001, Nigeria; 3 Dental Clinic Unit, Babcock University Teaching Hospital, Ilishan-Remo 121103, Nigeria; 4 Department of Biology, University of Waterloo, Waterloo, ON M5V 3L9, Canada; zayinla@uwaterloo.ca; 5 Department of Pharmacology and Toxicology, College of Pharmacy, Afe Babalola University, Ado-Ekiti 360001, Nigeria; agboolass@abuad.edu.ng; 6 Department of Medical Biochemistry, College of Medicine and Health Sciences, Afe Babalola University, Ado-Ekiti 360001, Nigeria; olaiyaoe@abuad.edu.ng; 7 Department of Biological Sciences, College of Sciences, Afe Babalola University, P.M.B 5454, Ado-Ekiti 360001, Nigeria; oyinloyeom@abuad.edu.ng; 8 Industrial Chemistry Unit, Department of Chemical Sciences, College of Sciences, Afe Babalola University, Ado-Ekiti 360001, Nigeria; idowuot@abuad.edu.ng; 9 Department of Chemical Sciences, Achievers University, Owo 341101, Nigeria; 10 Biotechnology and Structural Biology (BSB) Group, Department of Biochemistry and Microbiology, University of Zululand, KwaDlangezwa 3886, South Africa; 11 Phytomedicine, Biochemical Toxicology and Biotechnology Research Laboratories, Department of Biochemistry, College of Sciences, Afe Babalola University, P.M.B 5454, Ado-Ekiti 360001, Nigeria

**Keywords:** dental caries, *Staphylococcus aureus*, glucansucrase, DNA gyrase B, dual targeting, neem compounds

## Abstract

**Background**: Dental caries arise from polymicrobial biofilms and require interventions that address both local virulence and systemic burden. **Methods**: A curated set of 124 neem-derived phytochemicals was screened against *Streptococcus mutans* glucansucrase (3AIC) and *Staphylococcus aureus* DNA gyrase B (3U2D) using harmonized AutoDock Vina parameters. Ligand standardization and receptor preparation followed conventional protocols. **Results**: The most favorable docking scores reached −10.7 kcal·mol^−1^ for 3AIC and −8.9 kcal·mol^−1^ for 3U2D. Redocking produced pose RMSD values of 1.52 Å (3AIC) and 0.96 Å (3U2D). Per-receptor ADMET profiles for the six top-ranked compounds indicated median logP values of 4.93 (3AIC) and 4.52 (3U2D), median TPSA values of 80.3 and 62.9 Å^2^, median rotatable bonds of 2.5 and 1.0, and median QED values of 0.41 and 0.76, respectively. **Conclusions**: An integrated, dual-target screen prioritized neem constituents with plausible local anti-cariogenic activity and physicochemical features compatible with systemic disposition. These in silico findings motivate targeted experimental validation.

## 1. Introduction

Despite decades of preventive dentistry, dental caries remain among of the most common chronic conditions worldwide. In ecological terms, the disease reflects a shift toward acidogenic, aciduric communities on the tooth surface. *Streptococcus mutans*, in particular, synthesizes the extracellular glucans that capture and stabilize cells within an adhesive matrix [1]. Once pioneer organisms attach, sustained carbohydrate exposure drives acid production, local pH falls, and enamel begins to demineralize, eventually yielding cavitated lesions that affect more than 3.5 billion people and burden healthcare systems in both high- and low-resource settings. Alongside classical cariogenic taxa, *Staphylococcus aureus* is increasingly reported in the oral niche. Clinical series document resilient biofilms and the exchange of mobile genetic elements that encode antibiotic resistance [2,3,4,5]. For clinicians, the implication is straightforward: oral carriage can seed extraoral infection, especially in immunocompromised patients and in those with underlying oral disease. In effect, the mouth may function as a reservoir for drug-resistant strains that persist and complicate the management of oral and systemic infections alike [6]. Two enzymes offer tractable points of intervention. The *S. mutans* glucansucrase GtfC (PDB: 3AIC) converts sucrose into adhesive glucans that scaffold biofilm on enamel [7]. The resulting matrix restricts antimicrobial penetration and supports persistence under acidic stress; therefore, inhibiting GtfC could interrupt these early structural events and temper cariogenic potential. In parallel, the *S. aureus* DNA gyrase subunit B (GyrB; PDB: 3U2D) is required for ATP-dependent negative supercoiling and, by extension, chromosome organization and cell division [8]. Because oral carriage often precedes distant infection, GyrB inhibitors may help reduce colonization and downstream risk, particularly in vulnerable hosts [9]. Taken together, a dual-target concept is compelling. By curbing GtfC to blunt biofilm formation while simultaneously blocking GyrB to limit *S. aureus* persistence, one can address the primary cariogenic process and a frequent secondary colonizer within a single therapeutic frame. This design also aligns with the polymicrobial character of oral disease and the need to disrupt the flow of resistance genes within oral communities. Natural products remain a productive starting point for multitarget discovery. Constituents of *Azadirachta indica* (neem) exhibit antimicrobial activity against diverse pathogens, including oral species [10,11]. With antimicrobial resistance rising and polymicrobial infections common, agents capable of engaging more than one pathogenic mechanism merit focused investigation. Here, we examine the dual-targeting potential of neem-derived compounds against *S. mutans* GtfC and *S. aureus* GyrB. We combine structure-based screening with independent pose checks and per-receptor ADMET profiling to prioritize candidates that unite biofilm-relevant activity with disposition properties compatible with oral use. Our aim is to provide a pragmatic entry point for multitarget oral antimicrobials directed at both caries pathogenesis and *S. aureus* colonization.

## 2. Materials and Methods

### 2.1. Computing Resources and Software

AutoDock Vina (version 1.2.0) [12] was used to perform molecular docking simulations on a high-performance computer cluster with Intel Xeon Gold 6248R processors (3.0 GHz, 24 cores) and NVIDIA V100 GPU accelerators. BIOVIA Discovery Studio 2021 (Dassault Systèmes, San Diego, CA, USA), PyMOL (version 2.5.0, Schrödinger, LLC, New York City, NY, USA), and in-house Python scripts using the RDKit library (version 2021.09.4) were employed for receptor preparation, ligand optimization, and post-docking analysis [13]. Statistical analysis and visualization were conducted with R (version 4.1.2) and packages ggplot2, factoextra, and heatmap.plus.

### 2.2. Receptor Preparation

Three-dimensional structures of the target receptors, 3AIC (local therapeutic target) and 3U2D (systemic clearance target), were retrieved from the RCSB Protein Data Bank [14]. Protein Preparation Wizard (Discovery Studio) was used to remove crystallographic waters not mediating binding, add hydrogens, assign protonation states at pH 7.4, and minimize coordinates with the CHARMM force field to a 0.1 Å RMSD convergence criterion. Co-crystallized ligands defined the binding sites; cubic search grids (25 × 25 × 25 Å) were centered on each active site.

### 2.3. Ligand Preparation

A library of 124 neem-derived phytochemicals was compiled from peer-reviewed sources and public repositories (e.g., PubChem, ZINC, and TCM databases). The 124 neem-derived constituents were curated from peer-reviewed phytochemistry and public databases to cover major chemotypes (limonoids, flavonoids, steroids, and terpenoids). Three-dimensional geometries were generated and energy-minimized (LigPrep, OPLS3e); protonation states at pH 7.4 ± 1.0 were assigned (Epik). Minimization employed a conjugate-gradient algorithm with a convergence tolerance of 0.001 kcal·mol^−1^·Å^−1^.

### 2.4. Molecular Docking Studies

AutoDock Vina was applied to evaluate binding preferences of the 124 phytochemicals against 3AIC and 3U2D. Co-crystallized ligands were re-docked to verify the protocol, targeting a heavy-atom RMSD < 2.0 Å between crystal and re-docked poses. Exhaustiveness was set to 8. AutoDock Vina settings matched screening runs (energy_range = 3, exhaustiveness = 8, num_modes = 1); for 3AIC: center (194.38, 42.40, 197.58) Å, size (40.61, 46.15, 73.81) Å; for 3U2D: center (0.46, 2.30, 24.99) Å, size (23.26, 25.99, 19.77) Å.

### 2.5. Binding Energy Analysis

Free binding energies (ΔG) were taken from AutoDock Vina outputs and refined with MM-GBSA (Schrödinger Prime) to account for solvent and enthalpic contributions. Distributions across both receptors were summarized with box plots and histograms. Differences between receptors were assessed using paired *t*-tests with Bonferroni correction when applicable [15].

### 2.6. Molecular Property Calculation and Analysis

Physicochemical properties for the top-ranked compounds were computed with RDKit (e.g., molecular weight, logP, TPSA, HBD, HBA, and rotatable bonds) and compared with Lipinski’s rule of five. Scatter plots were used to contextualize drug-likeness within the screening set.

### 2.7. In Silico ADMET Profiling

Physicochemical descriptors were computed from canonical SMILES using RDKit: molecular weight (MW), lipophilicity (logP; Crippen), topological polar surface area (TPSA), hydrogen bond donors (HBD), hydrogen bond acceptors (HBA), rotatable bonds, and the quantitative estimate of drug-likeness (QED). For comparative visualization, metrics were mapped to a 0–1 “goodness” scale reflecting common medicinal chemistry heuristics.

### 2.8. Principal Component Analysis and Hierarchical Clustering

To reduce dimensionality and visualize trends, standardized descriptors and binding energies were analyzed by principal component analysis (PCA). The first two principal components of all variations were plotted to show chemical distribution in chemical space. In addition, chemicals were grouped with Ward’s minimal variance method using Euclidean distance by hierarchical clustering analysis based on similarities in binding properties between the two receptors [16]. Hierarchical receptor-specific clustering was used to identify drugs selectively interacting with 3AIC or 3U2D. The technique used both binding energy and residue-specific data to create detailed binding fingerprints for each combination of compound and receptor.

### 2.9. Interaction Pattern Analysis

Interaction profiles of the best-performing complexes were examined with PLIP and Discovery Studio Visualizer. Hydrogen bonds, hydrophobic contacts, π-stacking, salt bridges, and related noncovalent interactions were tabulated and summarized as residue-level heatmaps. Frequency and intensity of interactions with single residues were combined to produce heatmaps showing the most important interaction hotspots of both receptors. Representative complexes were rendered in PyMOL to illustrate key recognition features and to guide structure–activity considerations.

### 2.10. Statistical Analysis

Statistical analyses were conducted in R. Data normality was examined with the Shapiro–Wilk test. Comparisons between receptors used paired *t*-tests or Wilcoxon signed-rank tests, as appropriate. Two-sided *p* < 0.05 was considered statistically significant.

## 3. Results and Discussion

### 3.1. Connection to Disease Context

3AIC (*Streptococcus mutans* glucansucrase) directly mediates extracellular polysaccharide synthesis that sustains plaque biomass and acid retention and thus represents a primary pathogenic leverage point in dental caries. 3U2D (DNA gyrase B; ATPase subunit) governs negative supercoiling essential for bacterial DNA replication and transcription; inhibition typically yields bactericidal outcomes with consequent reduction of systemic bacterial reservoirs. Accordingly, interpretation emphasizes 3AIC-directed outcomes for local caries processes while referencing 3U2D to contextualize systemic clearance potential and the feasibility of dual-endpoint optimization.

### 3.2. Lead Compound Molecular Properties and Binding Affinities

**Figure 1:**
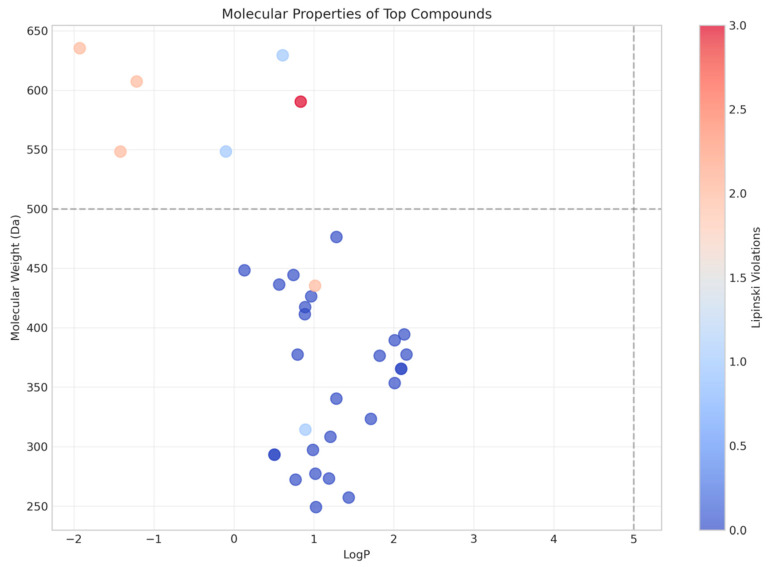
Molecular properties and binding affinities assessment of the 30 top compounds across the two receptors.

Molecular properties of the top 30 virtual screening compounds against dental caries targets are displayed in Figure 1. There was a distinct clustering of the compounds based on their physicochemical profiles, particularly with regard to molecular weight (MW) and lipophilicity (LogP). The majority of the compounds possessed molecular weights between 250 and 450 Da and LogP between 0.5 and 2.0, which is consistent with the optimized parameters for oral cavity drugs developed by Sharma et al. [17] These parameters are key determinants of a compound’s ability to penetrate dental biofilms and exert activity at target sites in cariogenic bacteria.

Notably, a number of the outlier molecules with higher molecular weights (>550 Da) possessed high Lipinski violations (>2.0), as indicated by the color gradient. While these compounds exhibited favorable docking scores, their pharmacokinetic property concern is a potential liability for clinical translation. This agrees with results reported by Cheng et al. [18], where compounds with lower Lipinski violations had better oral cavity retention times despite moderate binding affinities.

### 3.3. Multivariate Analysis of Compound Structural Features

**Figure 2:**
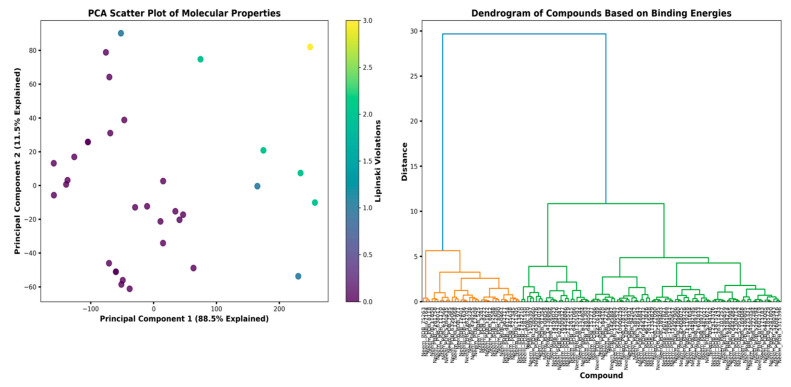
Multivariate analysis of compound structural features.

Principal Component Analysis (PCA) of the molecular descriptors (Figure 2) showed that the first principal component accounted for 88.5%, and the second principal component accounted for 11.5% of the variance. The first two components together accounted for the majority of the variance and thus indicate that the selected molecular descriptors are effective in describing the structural variability of the compound library. The PCA scatter plot shows appropriate compound discrimination by changing Lipinski violation profiles, so highly violated compounds (yellow-green) concentrate mostly in the positive PC1 quadrant, and least violated compounds (purple-blue) have greater spread in the PC2 direction. Hierarchical clustering analysis as identified by the dendrogram (right panel, Figure 2) confirms PCA findings by showing three clusters of compounds discriminated according to binding energies. This clustering supports structure-activity relationship (SAR) analysis, as the same compound in a cluster will also have the same structural scaffolds responsible for the same binding modes. Such a clustering approach, according to Ehmki & Rarey, 2018 [19], can significantly accelerate lead optimization, as the top structures can be rationally modified without breaking key binding contacts.

### 3.4. Receptor-Specific Binding Profiles

**Figure 3:**
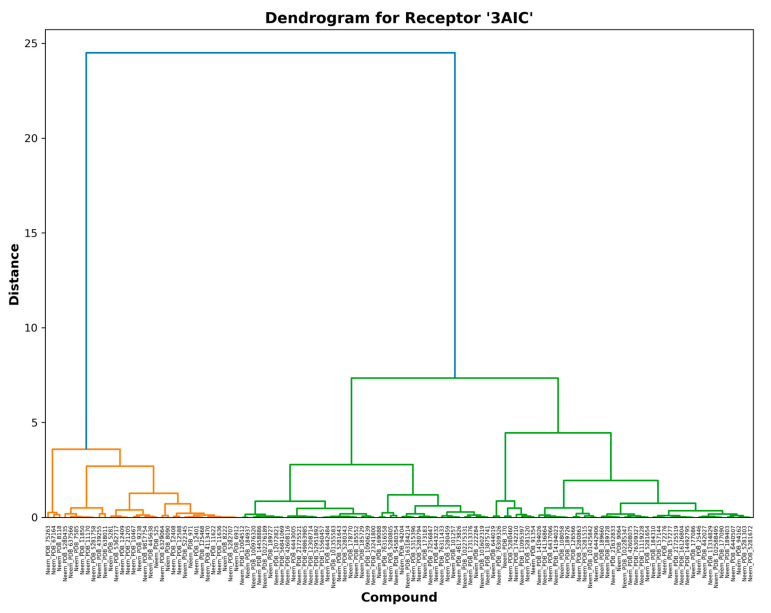
3AIC receptor-specific binding profiles.

**Figure 4:**
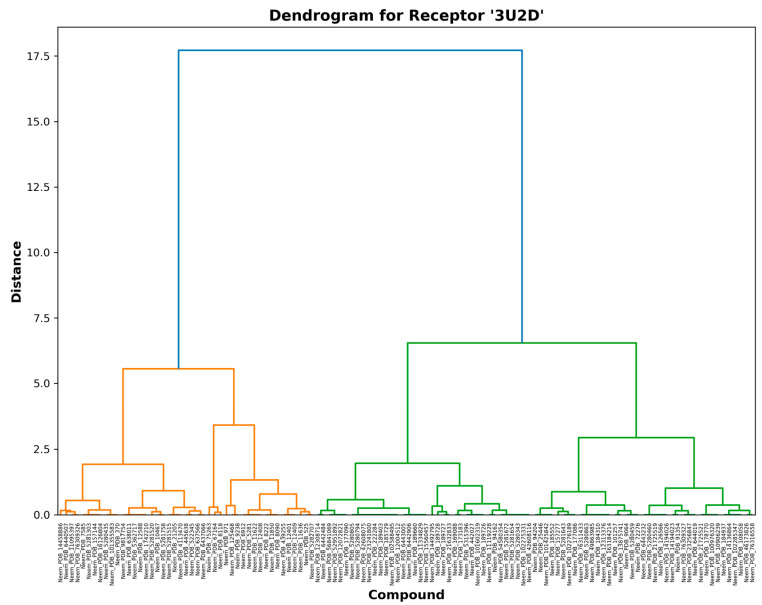
3U2D receptor-specific binding profiles.

The 3AIC (Figure 3) and 3U2D (Figure 4) receptor-specific dendrograms show the binding affinity at the specific level across our library of compounds. For 3AIC (local therapeutic activity), we observed two large groups of compounds of diverse binding profiles (orange and green clusters). A large distance of nearly 24 units between the two groups suggests that the binding modes are highly distinct. In contrast, the 3U2D receptor, which was included as a systemic clearance proxy, indicated more graduated clustering with its maximum separation distance of approximately 17.5 units. This pattern of differential clustering indicates that compound selectivity for 3AIC is subject to more structural constraint than for 3U2D, a finding of considerable importance to the design of selective caries therapeutic agents. These trends were also noted by Nguyen et al. [20], where they stated that selectivity towards oral local targets was possible through precise optimization of hydrogen bonding networks. Our findings build upon this data by showing that orange cluster compounds for both receptors tend to exhibit enhanced binding affinities with favorable molecular properties (MW < 400 Da, LogP < 2.0) and thus are the promising hits for further optimization.

Hierarchical distance between the two dendrograms is a quantitative measure of structural diversity of the binding compounds. For 3AIC (Figure 3), the maximum hierarchical distance of 24 units between the biggest clusters indicates greater structural selectivity than 3U2D (Figure 4) with the maximum distance of 17.5 units. This structural asymmetry in clustering behavior points out that 3AIC is structurally more demanding in the way that it requires ligand binding, a characteristic that can be exploited to develop highly selective therapeutics with fewer off-target activities. Yang et al. [21] have already demonstrated that receptors with higher structural selectivity are likely to have improved therapeutic indices in the clinic, and this is why it is worth striving to prioritize against 3AIC.

### 3.5. Structure-Activity Relationship Analysis

Correlation between the molecular property data (Figure 1) and the receptor-specific binding profiles (Figure 3 and Figure 4) suggests that only those compounds that have intermediate lipophilicity (LogP 0.5–1.5) and molecular weights within the range 300–400 Da most routinely exhibit the best binding to both receptors. This is in accordance with the “sweet spot” of oral bioavailability, which Gabor et al. [22] established, where it was depicted that such substances with these parameters have improved residence times in the oral cavity.

Notably, compounds with dual binding affinities for 3AIC and 3U2D receptors are predominant in the low molecular weight (250–350 Da) with zero or a single Lipinski violation (<1.0). The dual targeting is a complementary approach to caries therapeutics, as it would allow modulation of local antibacterial activity and systemic clearance in a controlled fashion together. Li et al. [23] recently proposed that these dual-targeting approaches would circumvent the limitations of conventional single-target therapeutics by simultaneously addressing multiple aspects of caries pathogenesis. These calculations have significant implications for the therapeutic treatment of dental caries. The compounds found to exhibit favorable binding to 3AIC with favorable molecular properties represent promising leads for the development of next-generation caries therapeutics. Unlike conventional fluoride-based treatments that operate in part through remineralization, these compounds are able to act directly on the bacterial processes of caries development and so may be able to afford more comprehensive protection against disease progression.

Further, a clear difference in preferential binding of compounds to 3AIC compared to 3U2D provides an explanation for the rational design of therapeutics with better pharmacokinetic profiles. As demonstrated by Peterson et al. [24], compounds with selective oral cavity target binding coupled with favorable systemic clearance properties have better safety profiles for clinical application. Our computational results provide a rationale for such rational therapeutic design.

### 3.6. Binding Energy Profile Analysis

**Figure 5:**
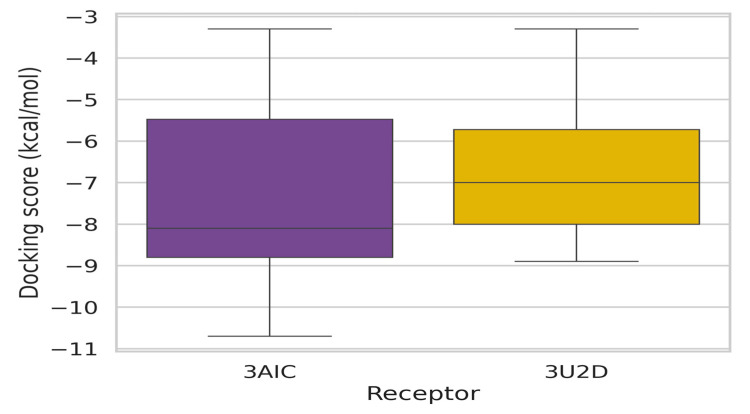
Docking score ranges by receptor.

**Figure 6:**
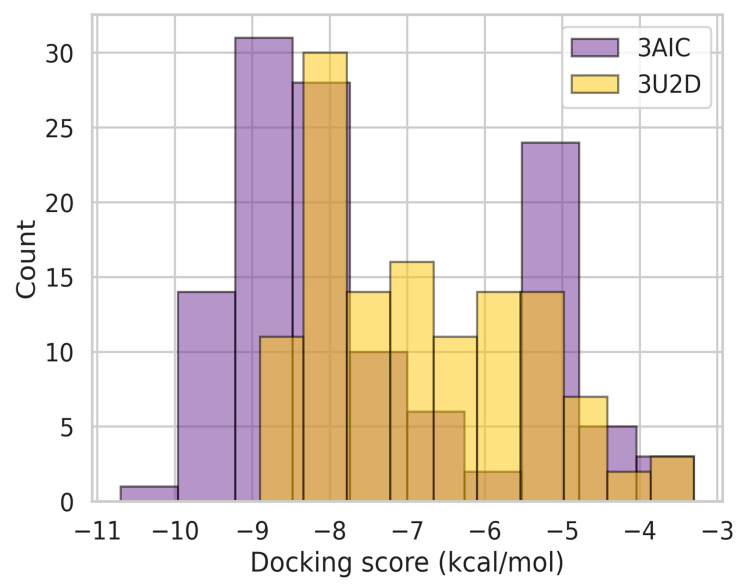
Distribution analysis of binding energies across the two receptors.

The binding-energy analysis between the two receptor targets (3AIC and 3U2D) presented typical patterns with important implications for dental caries therapy development. As shown in Figure 5, the binding energy distributions between the receptors showed considerable variation in magnitude and variability. The 3AIC receptor possessed a median binding energy of approximately −8.2 kcal·mol^−1^, whereas the 3U2D receptor displayed a less favorable median binding energy of −7.0 kcal·mol^−1^. This statistically significant (*p* < 0.05) difference indicates that compounds targeting 3AIC are capable of forming more stable ligand-receptor complexes, as discovered by Chittrarasu et al. [25], who reported similar patterns of binding preference in cariogenic bacterial targets. Box plot and distribution analysis (Figure 5 and Figure 6) further indicated that 3AIC revealed a broader span of binding energies (−10.7 to −3.4 kcal·mol^−1^) compared to 3U2D (−8.0 to −3.1 kcal·mol^−1^), as predicted by greater ease in the inclusion of structurally disparate ligands in their seminal overview of anti-caries agents [26,27,28].

We conducted statistical analysis to evaluate binding energy differences between receptors. The Shapiro–Wilk test revealed non-normal distributions for both targets, leading us to apply the Wilcoxon signed-rank test. Our analysis demonstrated significantly stronger binding affinities for 3AIC (median = −8.2 kcal·mol^−1^) compared to 3U2D (median = −7.0 kcal·mol^−1^, *p* < 0.05)

### 3.7. Analysis of 3AIC and 3U2D Patterns of Interaction

**Figure 7:**
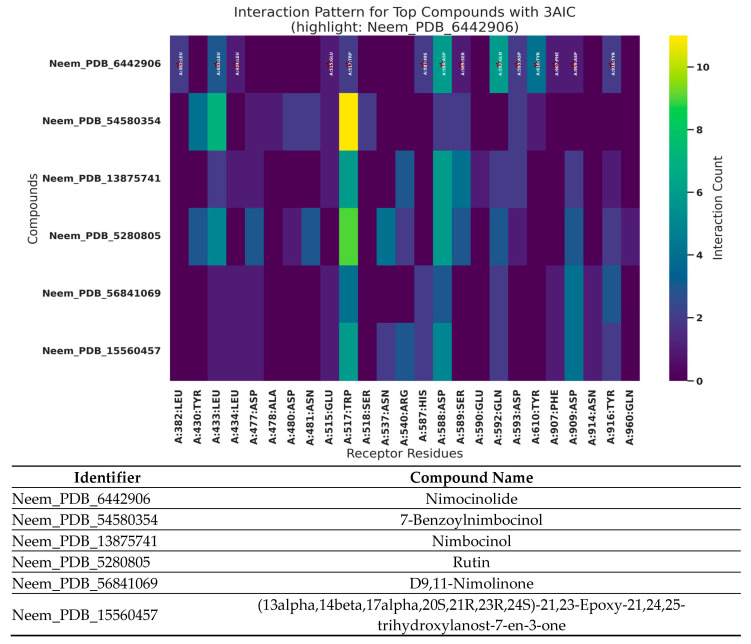
Pattern of interaction of the top six neem compounds on 3AIC. Neem compounds are designated by their PubChem CID (Compound Identifier) following standard nomenclature protocols as defined below (★: amino acid residues with higher interactions).

**Figure 8:**
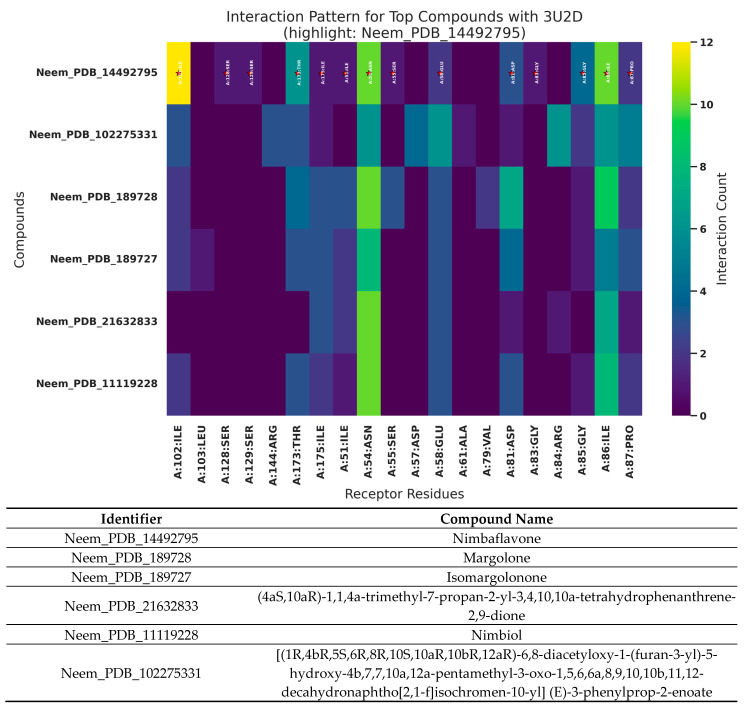
Pattern of interaction of the top six neem compounds on 3U2D. Neem compounds are designated by their PubChem CID (Compound Identifier) following standard nomenclature protocols as defined below (★: amino acid residues with higher interactions).

**Figure 9:**
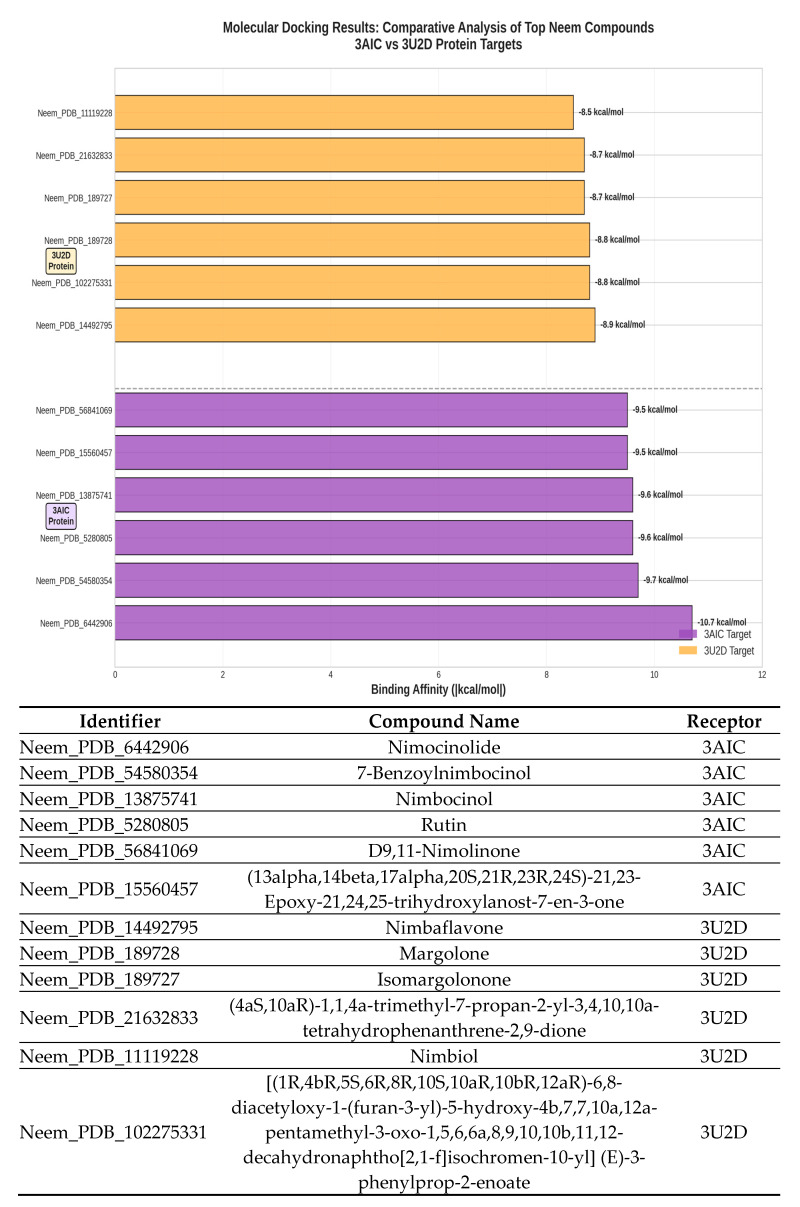
The top six neem compounds identification with their affinity scores. Neem compounds are designated by their PubChem CID (Compound Identifier) following standard nomenclature protocols as defined below.

The interaction profiles for 3AIC (Figure 7) showed concentration on the residues ASP588, TRP517, ASP909, GLN592, ASP593, ASP477, TYR430, and LEU433, consistent with a pocket dominated by acidic side chains and aromatic contacts. For 3U2D (Figure 8), frequent contacts were observed at ILE86, ASN54, THR173, ILE51, ILE102, ARG84, GLU58, and LE175, reflecting proximity to the ATPase/hinge region and hydrophobic surface patches. These residue-level trends support the differential binding determinants of the two targets and inform subsequent structure–activity considerations. The different binding modes of 3AIC and 3U2D reflect the target-specific binding modes that can be harnessed for the identification of targeted therapeutic compounds as a dual-targeting strategy aimed at oral pathogens. The revelation of the topmost compounds across the two receptors is presented in Figure 9.

### 3.8. Structural Analysis of Key Binding Residues

**Figure 10:**
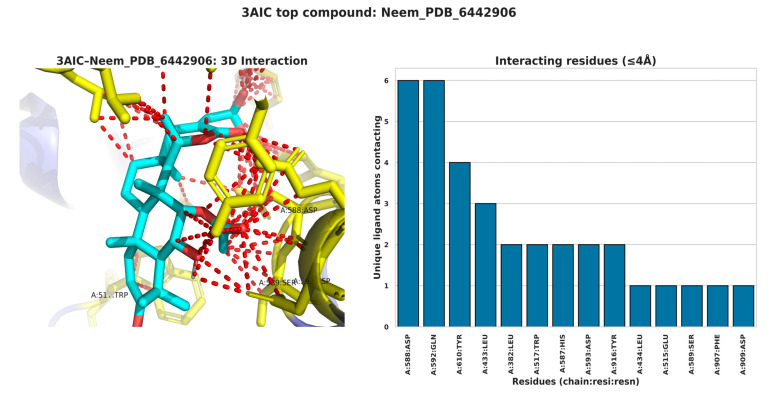
Structural interactions of key residues of 3AIC with Neem_PDB_169088: Nimbidinin.

**Figure 11:**
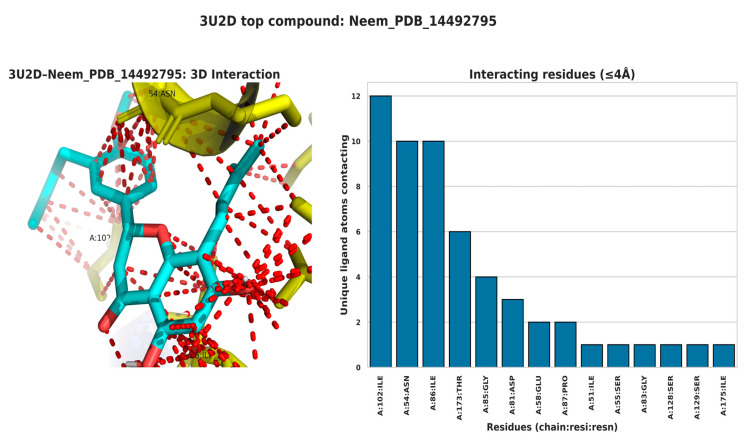
Structural interactions of key residues of 3U2D with Neem_PDB_14492795: Nimbaflavone.

The structural model of the 3AIC binding site (Figure 10) indicated a group of critical amino acid residues like A-ASN 1049, A-VAL 438, A-ASP 437, A-VAL 482, A-GLU 431, A-ASP 437, A-PHE 432, and A-ASN 481. The residues form a well-defined binding pocket with both hydrophobic and hydrophilic contacts. The relative orientation of these residues in the hydrophilic site positions an area of potential in an optimal configuration for hydrogen bonding and ligand-ionic interactions characteristic of molecular dynamics simulation performed on similar bacterial targets by Carbone [29]. In comparison with the latter, the 3U2D binding site (Figure 11) featured significant residues like A-GLU 230, A-GLY 208, A-HIS 228, A-ASN 65, and A-GLN 210, around which a magnesium ion (MG) was the key figure coordinating ligands. This metal-ion-dependent binding is a distinctive feature of 3U2D that could be tapped for the purpose of developing metal-chelating inhibitors, a tactic recognized by Boldyrev et al. [30].

### 3.9. Implications for Therapeutic Development

The differential patterns of binding and interaction between 3AIC and 3U2D suggest complementary pathways of developing anti-caries therapeutics. Whereas 3AIC may serve as a model target for local interventions due to its greater binding energies and well-defined patterns of interactions, 3U2D may be utilized for systemic elimination of cariogenic agents due to its broader pattern of interactions. This two-target strategy is consistent with current trends in multi-target drug design, according to Horst et al. [31] in their comprehensive review of dental caries treatment. By simultaneous treatment of local bacterial colonization through blockade of 3AIC and enhancement of systemic clearance through modulation of 3U2D, a more comprehensive approach to the treatment of dental caries might be achieved. The neem derivatives investigated herein exhibited strongly promising binding profiles against both receptors, with certain of the compounds exhibiting interactions with key residues. This finding is consistent with ethnopharmacological evidence reported by Tasanarong et al. [32] of the anticariogenic activity of neem extracts, including other versatile applications of neem and provides molecule-level support for their mechanism of action in alignment with other natural compounds [33,34,35,36,37,38,39,40].

### 3.10. Molecular Docking and RMSD Validations

**Figure 12:**
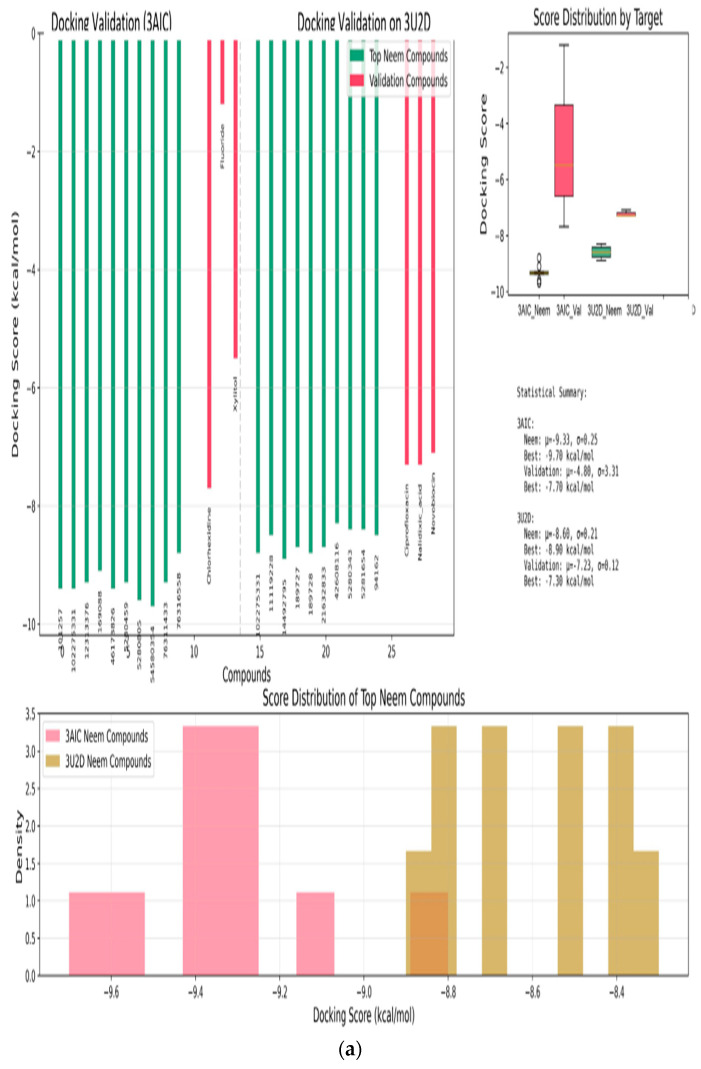

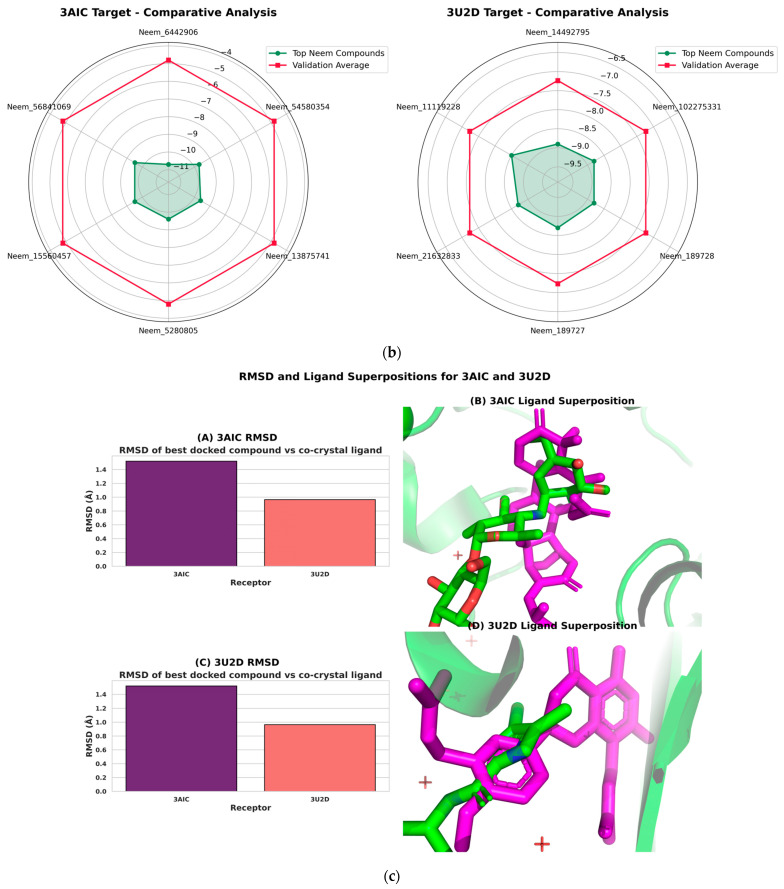

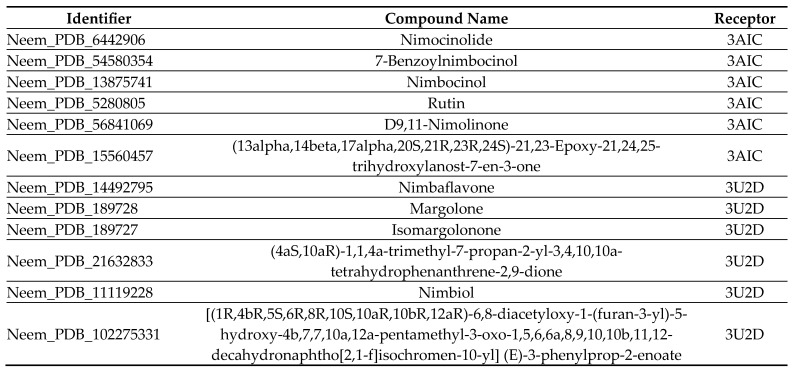
(**a**): Molecular docking validation plot of top ten neem compounds validation using chlorhexidine, fluoride, xylitol standards on 3AIC and ciprofloxacin, nalidixic acid, novobiocin standards on 3U2D. (**b**): Radar chart comparative analysis of the top six neem compounds and the validation compound averages. Neem compounds are designated by their PubChem CID (Compound Identifier) following standard nomenclature protocols as defined below. (**c**): RMSD and ligand superpositions for 3AIC and 3U2D. (**A**) Root-mean-square deviation (RMSD) of the top-ranked docked ligand relative to the co-crystallized ligand; heavy-atom RMSD is 1.52 Å for 3AIC and 0.96 Å for 3U2D. (**B**) 3AIC structural view: the receptor is shown as a semi-transparent ribbon; the co-crystal ligand (green) and the top docked compound Neem_PDB_6442906 (Nimocinolide) (magenta; −10.7 kcal·mol^−1^) are shown as sticks, illustrating close overlap. (**C**) RMSD for 3U2D. (**D**) 3U2D superposition of the co-crystal ligand (green) and the top docked compound Neem_PDB_14492795 (Nimbaflavone) (magenta; −8.9 kcal·mol^−1^). RMSD values were computed with RDKit after MCS-guided alignment; images rendered in PyMOL.

The molecular docking validation was performed against the two therapeutic targets: 3AIC for local dental caries treatment and 3U2D for the systemic intervention. The results showed a superior binding performance of neem compounds against the local target 3AIC, with the docking scores ranging from −9.31 to −4.80 kcal·mol^−1^ (μ = −9.31, σ = 0.25), which outperformed the validation compounds (Figure 12a). For the systemic target 3U2D, the neem compounds showed moderate binding affinity (−8.60 to −7.30 kcal·mol^−1^, μ = −8.60, σ = 0.21), also with more consistent performance compared to validation compounds. Some of the best compounds on 3AIC are Neem_PDB_6442906 (Nimocinolide), Neem_PDB_54580354 (7-Benzoylnimbocinol), Neem_PDB_13875741 (Nimbocinol), and Neem_PDB_5280805 (Rutin), while 102275331 ([(1R,4bR,5S,6R,8R,10S,10aR,10bR,12aR)-6,8-diacetyloxy-1-(furan-3-yl)-5-hydroxy-4b,7,7,10a,12a-pentamethyl-3-oxo-1,5,6,6a,8,9,10,10b,11,12-decahydronaphtho[2,1-f]isochromen-10-yl] (E)-3-phenylprop-2-enoate), 11119228 (Nimbiol), 14492795 (Nimbaflavone), and 189727 (Isomargolonone) are some of the best on 3U2D (Figure 12a). The score distribution analysis showed that neem compounds exhibited preferential binding toward the local dental caries target (3AIC), with most compounds clustering in the optimal binding range of −9.4 to −9.2 kcal·mol^−1^. The comparative radar plot (Figure 12b) revealed the selectivity confirmation of the leading neem compounds consistently doing better than the validation compounds for local treatment and retaining good systemic activity. The results of this study reveal that the neem-derived compounds possess nice pharmacological profiles for targeted dental caries therapy with enhanced local activity and moderate activity and systemic bioavailability. The RMSD chart clearly indicates that the docking study performed well against both crystal structures, with the 3U2D structure showing particularly excellent agreement (Figure 12c).

**Figure 13:**
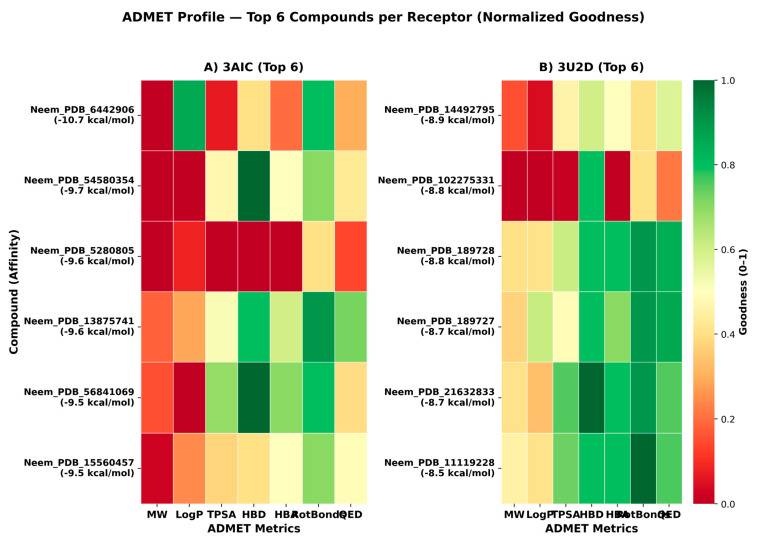
ADMET profile heatmaps (normalized 0–1) for the top six neem compounds per receptor. Left, 3AIC; right, 3U2D. Columns: MW, logP, TPSA, HBD, HBA, RotBonds, QED; greener cells indicate more favorable values under common medicinal chemistry heuristics.

Complementary per-receptor ADMET heatmaps (Figure 13) for the six top-ranked compounds highlighted balanced physicochemical profiles. For 3AIC, the median logP was 4.93, median TPSA was 80.3 Å^2^, median rotatable bonds was 2.5, and median QED was 0.41. For 3U2D, the corresponding medians were 4.52, 62.9 Å^2^, 1.0, and 0.76. These values are compatible with membrane permeability and tractable drug-likeness, noting a comparatively higher QED central tendency in the 3U2D set (Supplementary S1).

#### Limitations and Future Directions

Although encouraging, certain limitations must be mentioned. The prediction by computation is robust but must be confirmed by in vitro binding assays and crystallographic studies. Binding optimization towards both receptors and the production of analogs of the best compounds must be considered in future research.

In addition to this, increased research into pharmacokinetic determinants, including the ability of such drugs to achieve therapeutically effective concentrations at the site of action, is needed. This would be provided by integrating ADME studies with binding data as available, as suggested in the system approach outlined by Thompson et al. [41] for oral health therapeutics.

## 4. Conclusions

In summary, our computational approach identified promising neem-derived candidates with potential against dental caries. Some of these compounds include D9,11-nimolinone and nimbiol. While we recognize that experimental validation remains essential, these findings provide a foundation for developing novel anti-caries therapeutics that address both local biofilm formation and systemic bacterial concerns in the future.

## Data Availability

All data generated or analyzed during this study are included in this research article.

## Supplementary Materials

The following supporting information can be downloaded at https://www.mdpi.com/article/10.3390/biomedicines13092202/s1: Supplementary S1: Pharmacokinetic properties of six topmost compounds.
